# Insecticidal and genotoxic activity of *Psoralea corylifolia* Linn. (Fabaceae) against *Culex quinquefasciatus* Say, 1823

**DOI:** 10.1186/1756-3305-6-30

**Published:** 2013-02-04

**Authors:** Virendra K Dua, Arvind Kumar, Akhilesh C Pandey, Sandeep Kumar

**Affiliations:** 1 National Institute of Malaria Research, Sector-3, Health Centre, Field Unit BHEL, Ranipur, Hardwar, Uttrakhand, 249403, India; 2 Department of Biotechnology, Madhav Institute of Technology and Science, Gwalior, 474005 (M.P), India

**Keywords:** Larvicidal activity, Adulticidal activity, Genotoxicity, DNA damage, Essential oil, *Psoralea corylifolia*, *Culex quinquefasciatus*, GCMS

## Abstract

**Background:**

Indiscriminate use of synthetic insecticides to eradicate mosquitoes has caused physiological resistance. Plants provide a reservoir of biochemical compounds; among these compounds some have inhibitory effect on mosquitoes. In the present study the larvicidal, adulticidal and genotoxic activity of essential oil of *Psoralea corylifolia* Linn. against *Culex quinquefasciatus* Say was explored.

**Methods:**

Essential oil was isolated from the seeds of *P. corylifolia* Linn. Larvicidal and adulticidal bioassay of *Cx. quinquefasciatus* was carried out by WHO method. Genotoxic activity of samples was determined by comet assay. Identification of different compounds was carried out by gas chromatography- mass spectrometry analysis.

**Results:**

LC_50_ and LC_90_ values of essential oil were 63.38±6.30 and 99.02±16.63 ppm, respectively against *Cx. quinquefasciatus* larvae. The LD_50_ and LD_90_ values were 0.057±0.007 and 0.109±0.014 mg/cm^2^ respectively against adult Cx*. quinquefasciatus,*. Genotoxicity of adults was determined at 0.034 and 0.069 mg/cm^2^. The mean comet tail length was 6.2548±0.754 μm and 8.47±0.931 μm and the respective DNA damage was significant i.e. 6.713% and 8.864% in comparison to controls. GCMS analysis of essential oil revealed 20 compounds. The major eight compounds were caryophyllene oxide (40.79%), phenol,4-(3,7-dimethyl-3-ethenylocta-1,6-dienyl) (20.78%), caryophyllene (17.84%), α-humulene (2.15%), (+)- aromadendrene (1.57%), naphthalene, 1,2,3,4-tetra hydro-1,6-dimethyle-4-(1-methyl)-, (1S-cis) (1.53%), trans- caryophyllene (0.75%), and methyl hexadecanoate (0.67%).

**Conclusion:**

Essential oil obtained from the seeds of *P. corylifolia* showed potent toxicity against larvae and adult *Cx. quinquefasciatus*. The present work revealed that the essential oil of *P. corylifolia* could be used as environmentally sound larvicidal and adulticidal agent for mosquito control.

## Background

Mosquitoes are an important public health concern around the world. They not only cause nuisance to humans but also transmit several diseases like malaria, filaria, Japanese encephalitis, dengue fever, chikungunya [1] and yellow fever [2]. These diseases affect the health and quality of life of millions of people in subtropical and tropical countries [3]. Mosquitoes also cause allergic responses in humans that include local skin and systemic reactions such as angioedema [4]. In 2010, The WHO reported 216 million cases of malaria in the world with an estimated 6,55,000 malaria deaths [5]. An estimated 120 million people in tropical and subtropical areas of the world are infected with lymphatic filariasis [6]. Three billion people in the endemic areas are at risk of infection with Japanese encephalitis and incidence of the disease is 30,000–50,000 cases annually [7]. Over 40% of the world's population (approximately 2.5 billion) is at risk from dengue, WHO estimated 50–100 million dengue infections worldwide, annually [8]. Moreover, there are an estimated 200,000 cases of yellow fever (causing 30,000 deaths) worldwide annually [9]. *Culex quinquefasciatus* Say, 1823 (widely distributed mosquito in India) is a vector of important diseases, such as West Nile virus, filariasis, Japanese encephalitis, St. Louis encephalitis, avian malaria and bancroftian filariasis (*Wuchereria bancrofti*) [10]. *Cx. quinquefasciatus* is responsible for major public health problems in India with around 31 million microfilaraemics, 23 million cases of symptomatic filariasis, and about 473 million individuals potentially at risk of infection [11].

Synthetic insecticides were used extensively during the 1950s to control malaria in various countries by indoor residual spraying (IRS) as a larvicide [12]. Synthetic insecticides were also used to control adult mosquitoes by fogging [13]. The continuous use of synthetic insecticide such as malathion, DDT, HCH and deltamethrin for controlling mosquitoes has created diverse environmental problems such as toxicity to non target organisms [14], development of genetic resistance in mosquitoes [15], environment pollution [16] and their non degradable nature results in biomagnifications. Herein, the worldwide continuous efforts to eradicate and control this vector were found ineffective. Therefore, there is a need to search for environmentally safe, degradable and target specific insecticides. Plant derived essential oils are emerging as a potential source for mosquito control agents, since they constitute a rich source of bioactive compounds that are biodegradable and potentially suitable for controlling mosquitoes. Earlier researchers have reported the efficacy of several plant essential oils against mosquito larvae [17-21] and adults [22-26].

*Psoralea corylifolia* Linn. is an erect, herbaceous, and annual weed growing up to a height of 60–120 cm in the plains of central and eastern India, China, and in some parts of Arabia under semi arid conditions [27]. Seeds are usually brownish-black in color, smooth, and adhere to the pericarp [28,29]. The plant is widely used in several skin diseases such as psoriasis [30], leucoderma and leprosy [31]. The therapeutic action of *P. corylifolia* against various diseases such as asthama, diarrhoea, alopecia areata [32], impotency, menstruation disorder and uterine hemorrhage [33]. Moreover it has antitumor [34], antiallergic [35], antioxidant [36], insecticidal [37] and antimicrobial activity [38]. The plant has also been used for the treatment of enuresis, various kidney problems [39], depression [40], osteoporosis and bone fractures [27]. In the present study the larvicidal, adulticidal and genotoxic activity of volatile oil extracted from seeds of *P. corylifolia* against *Cx. quinquefasciatus* and the phytochemical analysis of volatile oil by GC-MS was determined.

The single cell gel electrophoresis (SCGE or Comet assay) is one of the most promising and imminent genotoxicity tests. It is less resource intensive than the usual genotoxic techniques and permits both qualitative and quantitative assessment of DNA damage in individual eukaryotic cells. The sensitivity of the SCGE technique has been applied in many areas, e.g. environmental monitoring [41], *in vivo* and *in vitro* genotoxicity testing [42] and epidemiological and biomonitoring studies in human populations exposed occupationally, environmentally or clinically [43,44]. This test procedure has been recommended in the Committee on Mutagenicity Guidelines of the UK Department of Health (COM) for determining *in vitro* mutagenicity of chemicals [45].

## Methods

### Plant material

Plants with seeds of *P. corylifolia* (Fabaceae) collected from Garhwal region of Himalaya were purchased from the Arya Vastu Bhandar Dehradun, Uttarakhand, India, which were further confirmed by Botanical Survey of India (BSI), Dehradun, India. The Voucher specimen of the whole plant and its seeds were kept in the Institute herbarium for future reference (Voucher Specimen No: NIMRHAR-101PC-1).

### Isolation of the essential oils

The collected seeds of *P. corylifolia* were washed with distilled water and dried under shade. The dried seeds (100g) were powdered with the help of a grinder and mixed in water (1:6); further steam distillation (in a Clevenger apparatus for 7 h), extracted the essential oil. The oil layer was separated from the aqueous phase using n-hexane with the help of a separating funnel. The volatile essential oil was dried using anhydrous sodium sulfate, and stored at 4°C until used.

### GC–MS analysis

The GC-MS analysis was carried out on a Shimadzu (QP 2010) series GC-MS (Tokyo, Japan) system equipped with AOC-20i auto-sampler and coupled with DB-5 MS capillary column (Agilent technologies, made in USA), (30 m × 0.25 mm i.d., 0.25 μm). Helium was used as carrier gas at a flow rate of 1.28 ml/min; split ratio of 1: 50; mass scan 50–800; ionization energy, 70 eV; ion source temperature, 200°C; injector temperature, 250°C. Oven temperature was programmed as follows: initially at 40°C for 5 min, rising at 4°C/min to 220°C and then held isothermally (5 min) at 220°C. The oil sample (10 μl) was diluted (up to 2 ml) with dichloromethane (HPLC grade), sample injection volume was 1 μl. Individual components were identified by comparison of their mass spectra (MS) with NIST database and Adams libraries from the derived fragmentation pattern [46,47].

### Test organisms

The test organism *Cx. quinquefasciatus* Say, 1823, was reared continuously from several generations in the Entomology Laboratory of the National Institute of Malaria Research, Field Unit, Hardwar, India. They were free of exposure to pathogens and insecticides and maintained at 26 ± 2°C and 60-80% relative humidity. The larvae were fed on dog biscuits and yeast powder in a ratio 3:2 until moulting to become pupae, pupae were transferred into a mosquito cage. The emergent adults were fed with 10% glucose solution dipped in a piece of cotton in humidified cages.

### Larvicidal bioassay

Larvicidal activities of the essential oil of *P. corylifolia* were determined in terms of LC_50_ and LC_90_ by using the standard procedure of WHO [48] with slight modification. Twenty early fourth instar larvae of *Cx. quinquefasciatus*, were transferred to 500 ml bowls containing 249 ml of dechlorinated tap water. The essential oil was dissolved in 1 ml acetone to prepare a serial dilution of test dosage and mixed in 249 ml tap water containing 20 early fourth instar larvae. Three replicates were run simultaneously with at least six dosages 25–100 μg/ml (ppm) along with control (1 ml of acetone alone to 249 ml of tap water). Bioassay was conducted at room temperature 26 ± 2°C with 60-80% relative humidity, during which time no food was offered to the larvae. Mortality of larvae was recorded 24 h post treatment and evaluated LC_50_ and LC_90_ by using probit analysis and StatusPlus2009 software.

### Adulticidal bioassay

Adulticidal bioassay was performed according to WHO guidelines [49] against *Cx. quinquefasciatus* and lethal dose (LD_50_ and LD_90_) and knockdown time (KDT_50_ and KDT_90_) were evaluated. Different concentrations ranges of the essential oil of *P. corylifolia* were prepared in 2.5 ml of acetone and homogenously applied on Whatman no. 1 filter papers (size 12 × 15 cm^2^), control papers were treated with 2.5 ml of acetone under similar conditions and placed in WHO exposure tubes. 20 adult mosquitoes (2–5 days old glucose fed mosquitoes) were exposed on treated paper for one hour and knocked down and live mosquitoes were counted at 5 minute intervals. After one-hour exposure mosquitoes was transferred into WHO holding test tubes for a 24 hour recovery period. During this period the mosquitoes were kept at room temperature at 26 ± 2°C and 70-80% relative humidity. The mortality was observed after 24 hours [50]. Three replicates were run simultaneously with at least six doses (0.034, 0.055, 0.069, 0.104, 0.138 and 0.173 mg/cm^2^) to produce a range of mortality from 15 to 100% along with controls. Lethal dose (LD_50_ and LD_90_) and knocked down time (KDT_50_ and KDT_90_) were calculated by probit analysis using StatusPlus2009 software.

### Positive control

Advance studies on adulticidal activity was carried out by using 0.05% deltamethrin (DM) impregnated paper as a positive control against female *Cx. quinquefasciatus* for determination of KDT_50_ and KDT_90_ value.

### Genotoxicity testing by comet assay

The DNA damage studies were carried out using Single Cell Gel Electrophoresis (SCGE), commonly known as comet assay. The protocol (Alkali method) was followed as described by Singh *et al.*[51] with minor modifications as described below.

### Slide preparation

Twenty mosquitoes were exposed to 0.034 mg/cm^2^ and 0.069 mg/cm^2^ concentrations of the essential oil of *P. corylifolia* as well as controls, for 1h in WHO exposure tubes and were homogenized in 10% (w/v) homogenizing buffer (0.075 M NaCl and 0.024 M EDTA). Homogenate was centrifuged at 1000 rpm for 10 minutes and the pellet was gently resuspended in 1ml of chilled homogenizing buffer for nuclei preparation. Frozen microscopic slides were then placed horizontally and a homogenous thin layer of 1% normal melting agarose was cast onto the slide, isolated nuclei and 1% low melting agarose [(1:4), 100 μl] were mixed and cast onto the precoated slides and kept at 4°C for 20 minute. The slides were immersed into the freshly prepared chilled lysis buffer (2.5 M NaCl, 100 mM EDTA pH 10, 5% DMSO, 1% and Triton-X 100) for 1 hour in the dark at 4°C. After complete lysis the slides were placed for 20 min in an ice cold electrophoresis chamber containing alkaline electrophoresis buffer (1mM EDTA and 300 mM NaOH, pH>13) to facilitate unwinding of DNA strands, the process was subsequently conducted for 20 minutes at 25 volts/300 mA. The slides were washed thrice with neutralizing buffer (0.4 M Tris pH 7.5) for 5 minutes and just before visualization. Slides were then stained with ethidium bromide (20 μg/ml, 40 μl/slide) for 10 min in the dark. Slides were then dipped once in chilled distilled water to remove excess ethidium bromide and subsequently cover slips were placed over them. The slides were stored in a dark, humidified chamber and analyzed within 3±4 h.

### Comet capture and analysis

A total of 100 cells from each slide were analyzed by image analysis using a fluorescence microscope (Leica DM4000B) with an excitation filter of 515–560 nm and a barrier filter of 590 nm using X10 objectives. The photographs of the individual cells were taken using a Leica Digital DFC 320R-II camera. Comet tail length and percentage of DNA damage in tail were measured with an Image Analysis System (Leica Qwin) and Comet Score software version 1.5 (TriTek Corporation, Sumerduck, VA).

### Statistical analysis

Statistical analysis of the experimental data was performed using the computer software StatPlus® 2009 (AnalystSoft, Canada) to find the lethal concentration/dose against larvae (LC_50_ and LC_90_) and adult (LD_50_ and LD_90_) in 24 h and also determines the knockdown time (KDT_50_ and KDT_90_) by probit analysis [52] with a reliability interval of 95%. To determine whether there was a statistically significant difference among different doses of *P. corylifolia* essential oils against mosquito larvae and adults, Student’s t-test was used to analyze the difference of the percentage of mortality. Results with P< 0.05 were considered to be statistically significant.

The corrected percent mortality was evaluated by using Abbott’s formula-

(1) $$\mathrm{Corrected}\;\mathrm{Mortality}\;\left(\%\right)\;=\;\frac{\left[\mathrm{MT-MC}\right]}{100-\mathrm{MC}}\times 100$$

where MT and MC are percent mortality in treated and control experiment, respectively [53].

## Results and discussion

The average yield of essential oil was 2% w/w according to their dry weight. Oil is a complex mixture of several compounds; chemical constituents of analyzed oils are displayed in Table 1.

**Table 1:** **Major identified compounds in the essential oil of *P. corylifolia *seeds by GCMS**

**S. No.**	**Retention time (min)**	**Concentration (%)**	**Constituents**	**Mass fragmentation pattern**	**Match with library**
1	30.500	17.84	caryophyllene	[M+] 204, 189,175,161, 147, 133, 120, 105, 93, 79, 69, 55, 51	NIST147.LIB
2	31.688	2.15	α-humulene	[M+] 204, 147, 121, 107, 93, 80, 67, 53	WILEY7.LIB
3	33.782	1.57	(+)- aromadendrene	[M+] 204, 187, 161, 147, 134, 119, 106, 96, 81, 69, 55, 51	WILEY7.LIB
4	33.842	1.53	naphthalene, 1,2,3,4-tetra hydro-1,6-dimethyle-4-(1-methyl)-, (1S-cis)	[M+] 202, 159, 144, 131, 105, 69	WILEY7.LIB
5	35898	40.79	(−)-caryophyllene oxide	[M+] 173, 164, 149, 135, 123, 107, 96, 79, 69, 55, 53	WILEY7.LIB
6	40.466	0.75	trans- caryophyllene	[M+] 236, 203, 189, 175, 161, 147, 133, 119, 105, 91, 79, 69, 55, 51	WILEY7.LIB
7	45.419	0.67	methyl hexadecanoate	[M+] 270, 239, 227, 199, 185, 171, 161, 143, 129, 119, 101, 87, 74, 69, 55	WILEY7.LIB
8	52.073	20.78	phenol,4-(3,7-dimethyl-3-ethenylocta-1,6-dienyl)	[M+] 256, 213, 185, 173, 158, 145, 127, 121, 107, 93, 83, 69, 55, 53	NIST147.LIB

### Phytochemical screening

A total of 20 compounds were identified in seeds of *P. corylifolia* essential oil. Compounds occurring in trace amounts are not reported in this article. Caryophyllene derivatives (caryophyllene oxide and caryophyllene) are major fractions of the essential oil (59.30%), among the caryophyllene derivatives, caryophyllene oxide and caryophyllene contributed 2/3 and 1/3 fraction, respectively. But 8 major compounds constituting 86.08% of the *P. corylifolia* seed essential oil were caryophyllene oxide, phenol, 4-(3,7-dimethyl-3-ethenylocta-1,6-dienyl), caryophyllene, α-Humulene, (+)- aromadendrene, naphthalene, 1,2,3,4-tetra hydro-1,6-dimethyle-4-(1-methyl)-, (1S-cis), trans- caryophyllene, and methyl hexadecanoate (Table 1). The results differ from Kapoor [54] and Sharma *et al.*[55] who reported that the main constituent of essential oil of *P. corylifolia* seeds have limonene, α-elemene, γ-elemene, β-caryophylenoxide, 4-terpineol, linalool, geranylacetate. We assume that the discrepancy might have been caused by the differences in the chemo types of the species. Several studies have shown that caryophyllene oxide and caryophyllene present in the essential oils of different plants possess significant insecticidal activities against different species of mosquitoes [56-59]. A range of essential oils exhibited bioactive properties against the larvae of *Cx. quinquefasciatus*[21,60-62].

### Larvicidal activity

**Figure 1:**
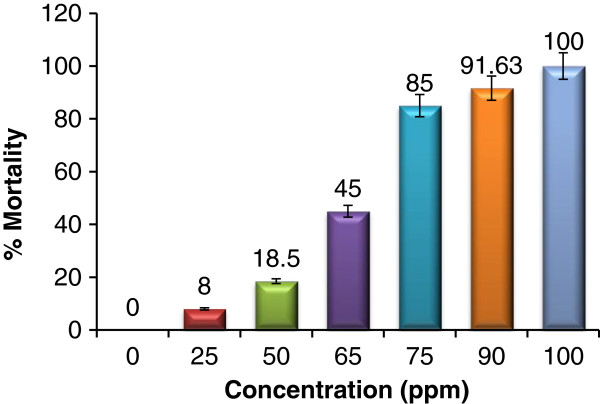
**Mosquito larvicidal activities of seed essential oil from *P. corylifolia *against *Cx. quinquefasciatus *larvae with significant concentration difference at p <0.05.**

In the present study larvicidal activity of essential oil from *P. corylifolia* was evaluated at different concentrations (range: 25–100 ppm) on early fourth instar larvae of *Cx. quinquefasciatus* and 8.0, 18.5, 45.0, 85.0, 91.63 and 100.0% mortality was recorded at 25, 50, 65, 75, 90 and 100 ppm, respectively (Figure 1). The data were analyzed using Student’s t-test in which p values <0.05 were taken to represent significant differences between mean values. Mean LC_50_ and LC_90_ (± standard error) values were 63.38±6.3 ppm and 99.02±16.63 ppm against *Cx. quinquefasciatus* larvae respectively (Table 2). In 2005, Dharmagadda and coworkers reported the larvicidal activity of *Tagetes patula* essential oil against the fourth instar larvae of *Aedes aegypti, Anophleles stephensi*, and *Cx. quinquefasciatus*, the LC_50_ and LC_90_ values were 13.57, 12.08, 22.33 and 37.91, 57.62, 71.89 ppm respectively [62]. In 2009, Pavela screened 22 essential oils for their larvicidal activity, the essential oil obtained from *Thymus vulgaris, Satureja hortensis* and *Thymus satureioides* plants found potent larvicidal activity with LC_50_ of 33, 36 and 44 g/ml, respectively against *Cx. quinquefasciatus*[60]. A series of plant essential oils were also reported as larvicide against different mosquito species [57,63].

**Table 2:** **Insecticidal activity of essential oil from seeds of *P. corylifolia *against *Cx. quinquefasciatus *larvae and adults**

**Larvicidal activity**		**Adulticidal activity**	
**LC**_**50**_**± SE**	**LC**_**90**_**± SE**	**LD**_**50**_**±SE**	**LD**_**90**_**± SE**
63.38±6.3	99.02±16.63	0.057±0.007	0.109±0.014

Six concentrations were tested with control (significantly different at P < 0.05) three replicates were taken. ^SE^Standard error, LC and LD values were determined by probit analysis.

### Adulticidal activity

A large number of plants are reported to have larvicidal activity, but very few of the plants are reported to have adulticidal activity [22,24,50].

**Figure 2:**
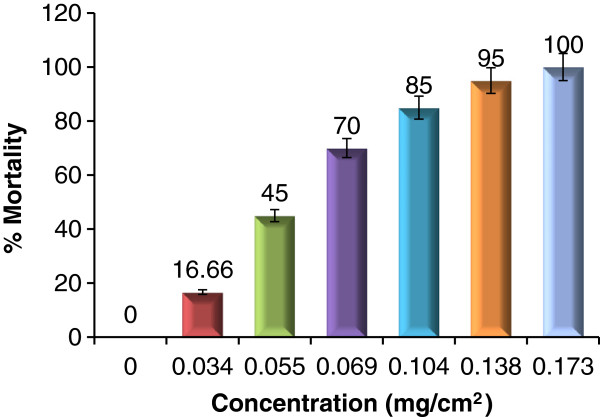
**Adulticidal activities of seed essential oils from *P. corylifolia *against adult female *Cx. quinquefasciatus *with significant differences of doses at p <0.05.**

**Figure 3:**
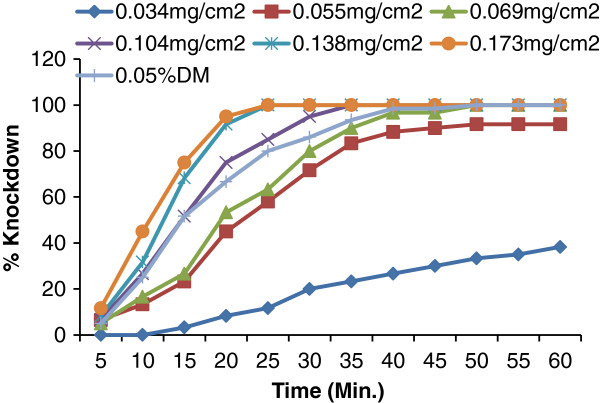
**The % Knockdown of female *Cx. quinquefasciatus *at different time intervals and different concentrations of seed essential oils from *P. corylifolia *with significant differences of doses at p <0.05.**

The present study demonstrates the adulticidal activity of essential oil from *P. corylifolia* at different concentrations (0.034, 0.055, 0.069, 0.104, 0.138 and 0.173 mg/cm^2^) on Whatman no.1 impregnated filter paper, against female adult *Cx. quinquefasciatus* mosquitoes and showed 16.66, 45.00, 70.00, 85.00, 95.00 and 100% mortality, respectively (Figure 2). The data were analyzed using Student’s t-test in which p values <0.05 were taken to represent significant differences between mean values. The LD_50_ and LD_90_ values (± standard error) were 0.057±0.007 mg/cm^2^ and 0.109±0.104 mg/cm^2^ against adult *Cx. quinquefasciatus* respectively (Table 2); the results were compared with 0.05% deltamethrin (DM) impregnated paper. KDT_50_ and KDT_90_ values of the essential oil from *P. corylifolia* were 20.29±0.88, 18.06±1.32, 13.45±0.60, 11.16±0.49, 9.87±0.48 min and 47.87±3.18, 36.00±1.32, 25.75±1.25, 19.40±0.96, 17.85±0.95 min respectively at 0.055, 0.069, 0.104, 0.138 and 0.173 mg/cm^2^ concentrations, respectively against *Cx. quinquefasciatus* (Figure 3). KDT_50_ and KDT_90_ values of 0.05% deltamethrin impregnated papers were 14.91±0.67 and 30.89±1.58 min, respectively against *Cx. quinquefasciatus,* with 96.7% mortality (Table 3). Dua and collaborators (2008) [50] reported the adulticidal activity of essential oil of *Valeriana jatamansi* root against *An. stephensi*, *An. culicifacies*, *Ae. aegypti*, *Ae. albopictus*, and *Cx. quinquefasciatus,* with LD_50_ and LD_90_ values were 0.14, 0.16, 0.09, 0.08, 0.17 mg/cm^2^ and 0.24, 0.34, 0.25, 0.21, 0.28 mg/cm^2^, respectively; Whereas KDT_50_ and KDT_90_ values were 13, 13, 12, 13, 18 min and 24, 25, 21, 20, 42 min against *An. stephensi*, *An. culicifacies*, *Ae. aegypti*, *An. albopictus* and *Cx. quinquefasciatus*, respectively, using 0.28 mg/cm^2^ impregnated papers. In 2010 Dua *et al.*[22], reported the adulticidal activity of essential oil of leaves of *Lantana camara* against *Ae. aegypti*, *Cx. quinquefasciatus, An. culicifacies, An. fluvialitis* and *An. stephensi,* LD_50_ values were 0.06, 0.05, 0.05, 0.05 and 0.06 mg/cm^2^ while LD_90_ values were 0.10, 0.10, 0.09, 0.09 and 0.10 mg/ cm^2^ respectively. Whereas KDT_50_ values were 20, 18, 15, 12, 14 min and KDT_90_ values were 35, 28, 25, 18, and 23 min against *Ae. aegypti, Cx. quinquefasciatus, An. culicifacies, An. fluviatilis* and *An. stephensi*, respectively on 0.208 mg/cm^2^ impregnated paper. Adulticidal activity of five essential oils (*Citrus sinensis, Mentha pipreta*, *Carvocryl* oil, *Citronela* oil and *citral* oil) at different concentrations and time intervals was determined by Yang *et al.* (2005), the Rutaceae oil (*C. sinensis*) was found as the most toxic against *Cx. quinquefasciatus* with LC_50_ of 0.0513 [24].

**Table 3:** **Mean knockdown time and corrected percent mortality of essential oil of *P. corylifolia *against adult *Cx. quinquefasciatus *at different concentrations**

**Concentration (mg/cm**^**2**^**)**	**% kd in 1 hr.**	**Kdt**_**50**_	**Kdt**_**90**_	**% Corrected mortality in 24 hr.**
0.034	38.3	ND	ND	20
0.055	91.65	20.29±0.88	47.87±3.18	45
0.069	100	18.06±1.32	36.00±1.32	70
0.104	100	13.45±0.60	25.75±1.25	85
0.138	100	11.16±0.49	19.40±0.96	95
0.173	100	9.87±0.48	17.85±0.95	100
Control	0	-	-	0
0.05% Deltamethrin	100	14.91±0.67	30.89±1.58	96.7

^Kdt^ Knockdown time (minutes)±standard error, ^ND^not determined. Six concentrations were tested with control (significantly different at P < 0.05) three replicates were taken. Kdt values were determined by probit analysis and mortality was corrected using Abbott's formula.

### Genotoxicity

**Figure 4:**
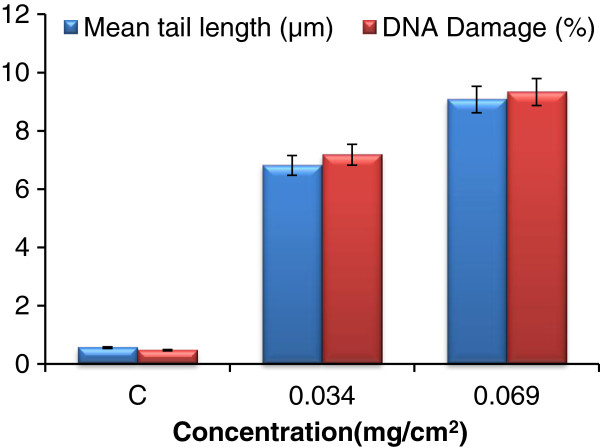
**Effect of seed essential oil from *P. corylifolia *on tail length and DNA Damage against female Cx. Quinquefasciatus.**

The effect of the of essential oil from *P. corylifolia* on DNA damage in individual cells of adult *Cx. quinquefasciatus* was assessed by two distinct types of DNA damage measurements: the length of DNA comet tail and the percentage of fragmented DNA present in the tail after electrophoresis. The DNA damage was deduced in adult *Cx. quinquefasciatus* when exposed to 0.034 mg/cm^2^ and 0.069 mg/cm^2^ concentrations of essential oil of *P. corylifolia* using the comet assay method. The observation showed that DNA damage was significant i.e. 6.713% and 8.864% at 0.034 mg/cm^2^ and 0.069 mg/cm^2^ concentrations of essential oil in comparison to controls (Figure 4). The comet tail length increases with increase in concentration of essential oil of *P. corylifolia*, the tail length was 6.2548±0.754 μm and 8.47±0.931 μm at 0.034 mg/cm^2^ and 0.069 mg/cm^2^ concentration respectively with reference to control, which confirms the genotoxicity (Figure 4). A literature study revealed that there was DNA damage in mid gut cells of third instar larvae of *D. melanogaster* on exposure to chlorpyrifos for 24 and 48 h, as assessed by comet assay. The tail length at 1.5 mg/l and 15.0 mg/l is 9.63 μm and 19.26 μm at 24 h, 9.87 μm and 28.21 μm at 48 h, respectively. The DNA damage was 9.65% and 18.94% at 24 h, 1.09 and 27.14% at 48 h, respectively [64].

The possible mechanism of essential oil toxicity is either to react with DNA or by the generation of ROS (reactive oxygen species) therefore causing DNA damage including in the adult *Cx. quinquefasciatus.* ROS are generated by inhibition of mitochondrial ATP synthesis through the uncoupling of oxidative phosphorylation that could lead to the generation of ROS [64,65]. During normal metabolism of the cell, ROS are generated in very low amounts and regulate various biological processes such as signal transduction pathways. At high and/or sustained levels, they can cause severe damage to DNA, protein and lipids [66]. Various stressors present in the environment including pesticides are capable of reacting with DNA and causing DNA damage, stressors also have the capability to generate ROS, one of the possible mechanisms for the induction of DNA damage may be through the generation of ROS [67].

## Conclusion

In the present study essential oil obtained from the seeds of *P. corylifolia* has shown potent toxicity against larvae and adults of *Cx. quinquefasciatus*. GC–MS analysis revealed the major constituents of essential oils were caryophyllene oxide, phenol,4-(3,7-dimethyl-3-ethenylocta-1,6-dienyl) and caryophyllene. The findings may be utilized for the development of eco-friendly insecticide, which could be used as an alternative for mosquito control.

## Competing interests

The authors declare that they have no competing interest.

## Authors’ contributions

VKD designed the work and supervised the manuscript. AK performed experiments, interpretation of data and drafted the manuscript ACP performed entomological studies. SK Analysis of data and experimental work. All authors read and approved the final version of the manuscript.
